# Use of methotrexate in an exuberant case of confluent and reticulated papillomatosis of Gougerot and Carteaud in a teenager^☆^^☆☆^

**DOI:** 10.1016/j.abd.2018.09.002

**Published:** 2019-10-26

**Authors:** Alexandra Brugnera Nunes de Mattos, Carolina Finardi Brummer, Gabriella Di Giunta Funchal, Daniel Holthausen Nunes

**Affiliations:** a Dermatology Service, Hospital Universitário Polyodoro Ernani de São Thiago, Universidade Federal de Santa Catarina, Florianópolis, SC, Brazil; b Pathology Service, Hospital Universitário Polyodoro Ernani de São Thiago, Universidade Federal de Santa Catarina, Florianópolis, SC, Brazil

**Keywords:** Diagnosis, Hyperpigmentation, Papilloma, Pathology

## Abstract

Confluent and reticulated papillomatosis of Gougerot and Carteaud is a rare dermatosis with onset during puberty, more prevalent in females than in males. The pathogenesis is unknown, but some theories suggest either a keratinization or endocrine disorder. The lesions are verrucous, brownish, hyperkeratotic papules or spots that coalesce in a confluent and/or reticulated pattern. This report presents a case with extensive cutaneous involvement associated with acanthosis nigricans and good response to treatment with methotrexate.

## Introduction

Confluent and reticulated papillomatosis (CRP) of Gougerot and Carteaud is characterized by the presence of brownish, verrucous papules with a diameter ranging from 1 to 5 mm, centrally confluent and peripherally reticulated, with the formation of plaques with imprecise borders, usually scaly. The eruption is asymptomatic or mildly itchy.^1^, ^2^, ^3^ The authors present an exuberant case of CRP, associated with acanthosis nigricans (AN), with good response to treatment with methotrexate.

## Case report

**Figure 1:**
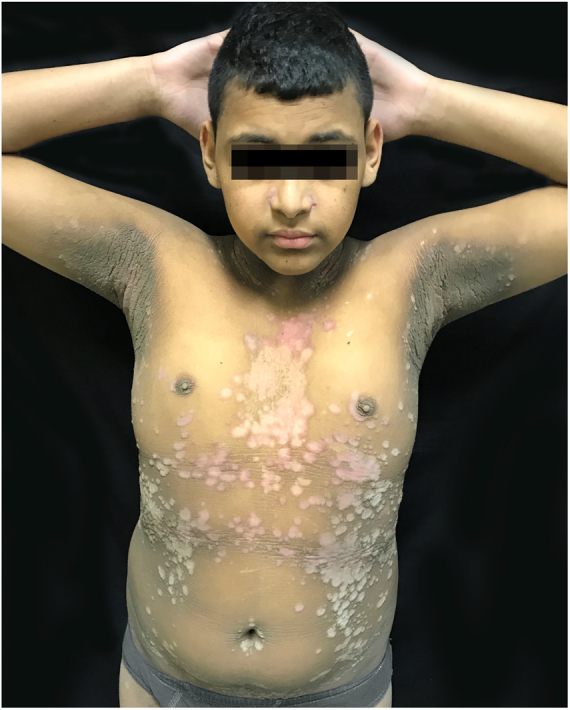
Erythematous, verrucous plaques, distributed on the trunk and abdomen.

**Figure 2:**
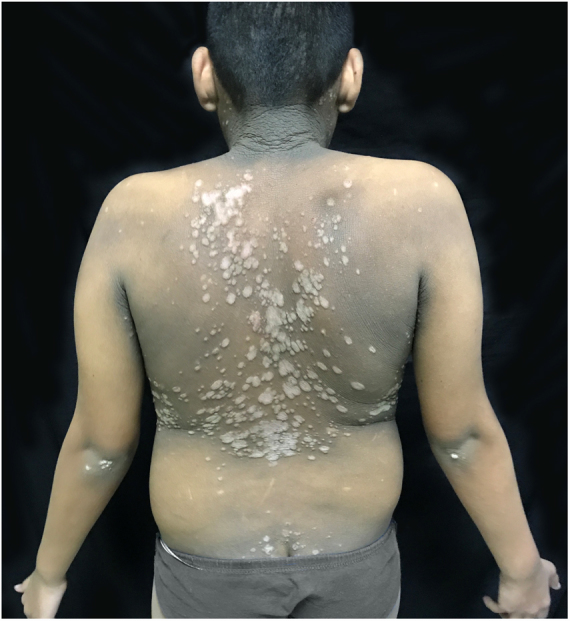
Erythematous, verrucous plaques, distributed on the trunk and abdomen.

**Figure 3:**
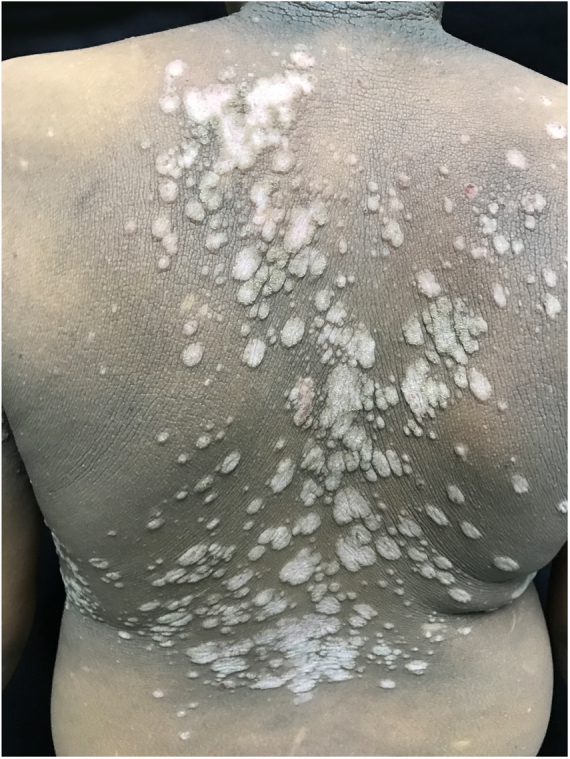
Details of the lesions.

**Figure 4:**
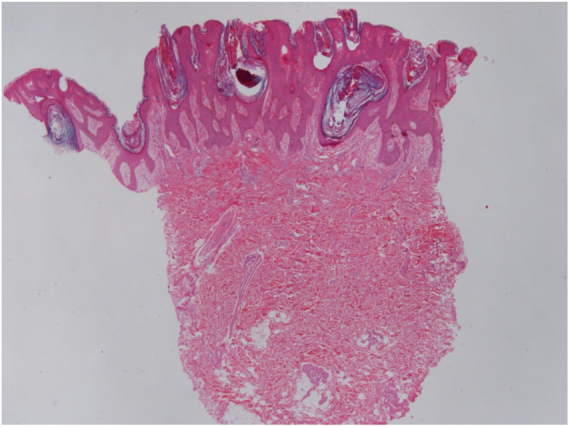
Skin sections showing a papillomatous surface covered by lamellar corneal layer with numerous spores of *Malassezia* spp. (Hematoxylin & eosin, x40)

**Figure 5:**
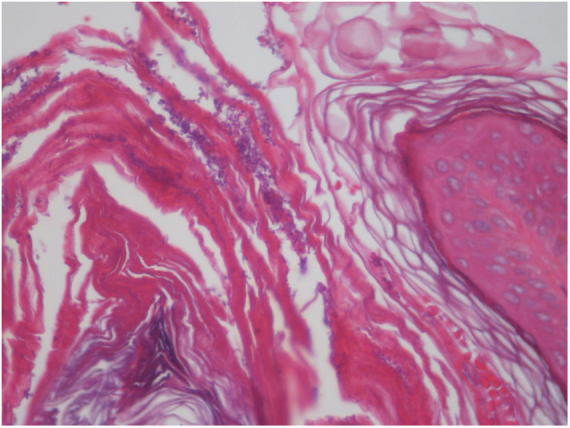
*Malassezia* spp. spores in the stratum corneum (Hematoxylin & eosin, x400) .

**Figure 6:**
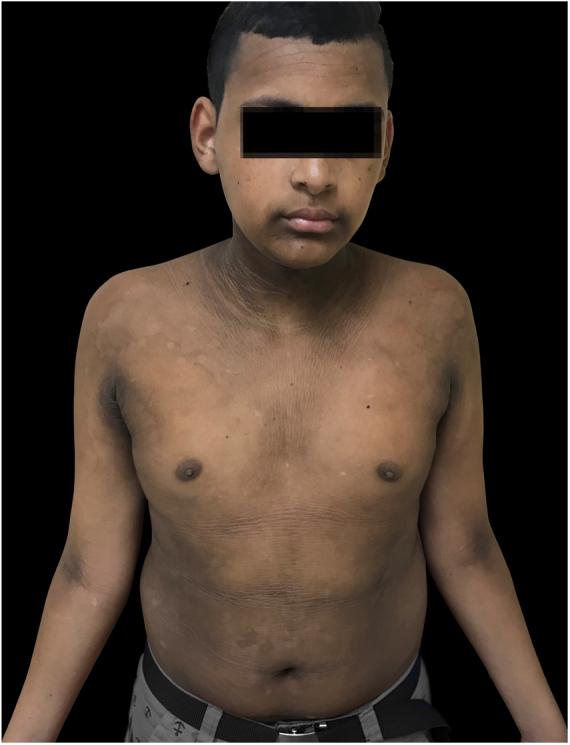
Outcome after treatment, improvement of lesion's appearance.

The patient was a 13-year-old boy with a history, starting at five years of age, of hyperchromic, scaly lesions, mildly pruritic, with a fetid odor, located on the trunk, abdomen, scalp, and cervical and axillary regions. He had received several previous treatments with acitretin 10 mg/day and topical medications such as retinoids, salicylic acid, and urea, without satisfactory results. The patient was undergoing treatment for a depressive mood disorder (sertraline 50 mg/day and amitriptyline 25 mg/day). Dermatological examination revealed brownish plaques with a verrucous and scaly surface, confluent, and with a reticulated pattern, distributed on the anteroposterior trunk and scalp (Figure 1, Figure 2, Figure 3). He also had hyperchromia and dark-brown velvety plaques in the axillary and cervical regions. Biopsies were performed in the interscapular region, showing a thick corneal layer with numerous spores of *Malassezia* spp., as well as melanotic hyperpigmentation of the basal keratinocytes (Figure 4, Figure 5). Thus, the diagnosis was established of CRP of Gougerot and Carteaud on the trunk and AN on the intertriginous areas. A detailed investigation was performed for other comorbidities, but he did not present hepatic, renal, or thyroid alterations, besides showing normal values for blood glucose, protein electrophoresis, serologies for hepatitis B and C, HIV, and syphilis, as well as cholesterol and triglycerides, and was only diagnosed with lactose intolerance. Abdominal ultrasound revealed Grade I hepatic steatosis. Upper gastrointestinal endoscopy showed no alterations, and he tested negative for *H. pylori*. The initially proposed treatment was dietary change, fluconazole 300 mg/week for three weeks, doxycycline 100 mg/day for one month, plus moisturizing cream, 10% glycolic acid lotion, and a cream containing 40 mg of hydroquinone, 0.5 mg of tretinoin, and 0.1 mg of fluocinolone acetonide on the axillary and cervical regions. The patient returned after one month with partial improvement of the lesions and liver function tests all within the normal range for age. Therefore, it was decided to maintain fluconazole 300 mg/week and topical medications as previously described, in addition to starting methotrexate 15 mg/week and folic acid 5 mg/week. The patient returned two months later with a significant reduction in the number of lesions and significant emotional improvement (Fig. 6).

## Discussion

CRP presents hyperpigmented, asymptomatic, verrucous plaques with peripheral reticulation, mainly in the cervical and axillary regions and trunk.^1^, ^2^, ^3^ Dermoscopy can be used to evaluate CRP, but diagnosis is essentially clinical.^4^, ^5^ The pathogenesis remains unknown, but some theories have suggested a disorder of keratinization due to the positive response to treatment with retinoids.^1^ Another suggested etiology is endocrine imbalance based on the insulin resistance found in some cases, as well as an association with obesity, diabetes mellitus, and other disorders of the pituitary and thyroid. An abnormal response to the growth of lipophilic yeasts of the species *Malassezia furfur* has also been suggested, since the proliferation of this fungus has been shown to be increased in the skin microbiota of patients with CRP.^1^ In the present case, exuberant growth of *M. furfur* was found in the biopsy, corroborating the CRP hypothesis. Differential diagnoses include Darier's disease, AN, pityriasis versicolor, and pseudoacanthosis nigricans.^1^, ^2^, ^5^ The present case presented AN in association with CRP, with thicker and more velvety plaques, without reticular pattern, on the axillary and cervical areas, typical of AN, and lesions consistent with CRP on the trunk. The literature has reported numerous treatment options for CRP, among which minocycline and azithromycin have shown good results, but additional options including other antibiotics, isotretinoin, acitretin, and antifungals comprise the therapeutic armamentarium.^1^, ^5^, ^6^ Spontaneous resolution of CRP without any treatment has been reported, and it has also been reported with weight reduction.^4^ However, opting for conservative treatment in patients with CRP is largely limited by the aesthetic and psychological distress it causes.^5^ Although no previous treatment of CRP with methotrexate was found in the literature, the drug was prescribed for this patient, obtaining good results after monotherapy and follow-up. Methotrexate is a folic acid antagonist with antiproliferative effects, preventing DNA synthesis and thus inhibiting tumor cell division in hematopoietic, mucosal, and other rapidly proliferating cells.^7^, ^8^, ^9^ In addition to being inexpensive, it has been used successfully in dermatology for the past 40 years because of its anti-inflammatory properties, mainly due to high adenosine levels, which inhibit neutrophil chemotaxis, prevent oxidative burst, improve cell barrier function in the endothelial cells, and inhibit the secretion of proinflammatory cytokines by monocytes and macrophages.^8^ Side effects of the drug include myelosuppression, mucositis, pain, diarrhea, vomiting, elevated hepatic transaminases, and rarely hepatotoxicity, teratogenicity, alopecia, acute renal failure, and pulmonary toxicity.^7^, ^8^, ^9^ It is an immunosuppressive agent with a strong safety and efficacy profile for various diseases (such as moderate to severe psoriasis, cutaneous T-cell lymphoma, collagenosis, vasculitis, atopic dermatitis, bullous diseases, and disseminated annular granuloma, among others) and offers the advantage of many years of experience in handling its toxicity and side effects. Further research on its mechanism of action and individual enzymatic variability may allow its use to be extended to other diseases, such as CRP.^8^ This report demonstrates that in addition to antibiotics, antifungals, and retinoids, methotrexate may be an option for the treatment of exuberant CRP. Despite the successful effect of methotrexate in this report, there remains a need for randomized controlled trials to establish the drug as an effective treatment for CRP.

## Financial support

None declared.

## Author's contribution

Alexandra Brugnera Nunes de Mattos: Conception and planning of the study; elaboration and writing of the manuscript; obtaining, analyzing and interpreting the data.

Carolina Finardi Brummer: Elaboration and writing of the manuscript; obtaining, analyzing and interpreting the data.

Gabriella Di Giunta Funchal: Approval of the final version of the manuscript; effective participation in research orientation; critical review of the literature; critical review of the manuscript.

Daniel Holthausen Nunes: Effective participation in research orientation; intellectual participation in propaedeutic and/or therapeutic conduct of the cases studied; critical review of the literature; critical review of the manuscript.

## Conflicts of interest

None declared.
